# Clinical Efficacy Analysis of Unilateral Modified Arytenoidectomy for Bilateral Vocal Fold Paralysis

**DOI:** 10.1002/wjo2.70006

**Published:** 2025-03-11

**Authors:** Lu Xing, Shu‐Yi Zeng, Pei‐Yun Zhuang

**Affiliations:** ^1^ Department of Voice Medicine Zhongshan Hospital of Xiamen University, School of Medicine, Xiamen University, Key Laboratory of Voice Medicine, Xiamen Science and Technology Bureau Xiamen China; ^2^ The School of Clinical Medicine Fujian Medical University Fuzhou China

**Keywords:** acoustic analysis, bilateral vocal fold paralysis, dyspnea, partial arytenoidectomy

## Abstract

**Purpose:**

To explore the clinical efficacy of unilateral modified arytenoidectomy (UMA) in the treatment of bilateral vocal fold paralysis(BVFP) based on animal models.

**Methods:**

The UMA which had been tested in excised canine larynges with simulated BVFP was adapted into a clinical technique. The characteristics of this surgical technique involve contouring of the arytenoid cartilage. The procedure expands the vocal fold respiratory region and subglottic space of the arytenoid cartilage while preserving the muscular process to maintain partial vocal fold muscle function. A retrospective analysis was conducted on 19 patients diagnosed with BVFP who were admitted to the Department of Voice Medicine, Zhongshan Hospital of Xiamen University between April 2019 and November 2023. These patients underwent CO_2_ laser arytenoid cartilage partial resection using a modified surgical technique. Dyspnea scores, voice handicap index scale (VHI‐10), and subjective and objective acoustic analysis were collected pre‐op and post‐op assessments to evaluate the clinical efficacy.

**Results:**

Data analysis of patients at 6 months post‐op showed VHI‐10: pre‐op (21.68 ± 12.40) score, post‐op (16.21 ± 7.68) score (*p* = 0.033); mMRC Dyspnea Scale: pre‐op (2.79 ± 0.71) score, post‐op (0.58 ± 0.69) score (*p* < 0.001); fundamental frequency (*F*0): pre‐op (175.54 ± 50.72) Hz, post‐op (190.36 ± 39.28) Hz (*p* = 0.196); maximum vocalization time (MPT): pre‐op (4.69 ± 4.30) s, post‐op (5.98 ± 3.24) s (*p* = 0.098); Jitter: pre‐op (6.12 ± 6.14)%, post‐op(2.39 ± 3.77)% (*p* = 0.090); Shimmer: pre‐op (22.27 ± 11.29)%, post‐op (13.02 ± 6.71)% (*p* = 0.048); Grade (G): pre‐op (2.56 ± 0.73), post‐op (1.78 ± 0.44) (*p* = 0.008); roughness (R): pre‐op (2.44 ± 0.73), post‐op (1.67 ± 0.50) (*p* = 0.020); breathiness (B): pre‐op (2.11 ± 1.05), post‐op (1.67 ± 0.50) (*p* = 0.102*)*; asthenia (A): pre‐op (1.33 ± 0.87), post‐op (1.00 ± 0.00) (*p* = 0.257); and strain (S): pre‐op (1.44 ± 0.88), post‐op (0.56 ± 0.73) (*p* = 0.046).

**Conclusions:**

The oral CO_2_ laser UMA is safe, minimally invasive, and highly effective. Postoperative voice quality shows significant improvement compared to the preoperative state. Moreover, the postoperative extubation rate can reach 100%, striking a balance between improving ventilation and preserving voice function to a considerable extent.

## Summary

- The results of previous animal model experiments have been translated into clinical techniques.
- The modified and innovative unilateral arytenoidectomy has been validated to relieve the dyspnea while maintaining the voice quality.
- The modified arytenoidectomy is minimally invasive with less postoperation complication.

## 1 Introduction

Bilateral vocal fold paralysis (BVFP) is a clinical condition characterized by airway obstruction, with symptoms including hoarseness, dyspnea, and obstructive sleep apnea hypopnea syndrome. The severity of dyspnea depends on the position of vocal folds, which is influenced by factors such as the type of laryngeal nerve injury, muscle function, muscle fibrosis, and the degree of stiffness of the cricoarytenoid joints. The objective of surgery for BVFP is to relieve the dyspnea while maintaining the voice quality as much as possible. Utilizing traditional surgical techniques, the animal models were established through four distinct surgical approaches to compare their impact on glottic airflow and voice quality [1]. To achieve better respiratory function while minimizing voice disorders, a method intermediate between the second and third surgical approaches was selected (Figure 2). This innovation of unilateral modified arytenoidectomy (UMA) was translated into clinical practice. This study aims to provide a preliminary exploration of the clinical efficacy of this innovative surgical technique.

## 2 Methods

This retrospective study analyzed 21 cases of BVFP treated at the Department of Voice Medicine, Zhongshan Hospital of Xiamen University from April 2019 to November 2023. All patients underwent UMA under microscopic laryngoscopy using a CO_2_ laser. The causes of BVFP included thyroidectomy, ataxia, idiopathic, and esophageal surgery. Among these, two patients had undergone tracheostomy before surgery, and eight patients had tracheostomy during the procedure. Two patients lost to follow‐up were excluded, leaving 19 patients (5 males, 14 females) with an age range of 42–71 years (mean age [53.47 ± 7.32] years). Detailed clinical information is provided in Table 1.

**Table 1:** Baseline characteristics of the studied patients.

Parameters	N = 19
Gender (*n*, %)	
Male	5 (26.3)
Female	14 (73.7)
Age (years)	
Mean ± SD	53.47 ± 7.32
Range	42–71
Cause of BVFP (*n*, %)	
Thyroidectomy	16 (84.2)
Esophageal surgery	1 (5.3)
Neck trauma	1 (5.3)
Idiopathic	1 (5.3)
Tracheostomy	
Tracheostomized	10 (52.6)
Non‐tracheostomized	9 (47.4)
Duration of metal tracheostomy tube placement (day)	
Mean ± SD	37.90 ± 26.38
Range	14–100

### 2.1 Preoperative Assessment

Evaluating the cause and duration of BVFP was performed for all enrolled patients; all of them had a history of progressive dyspnea associated with long‐term BVFP (ranging from 6 to 240 months). All patients underwent laryngostroboscopy, neck and chest CT imaging to exclude subglottic airway stenosis and to assess vocal fold mobility. Subjective voice assessment was performed using the GRBAS scale and the Voice Handicap Index 10 (VHI‐10) [2, 3]. Objective acoustic analysis was performed using lingWAVES v3.2.7 to evaluate voice quality and the modified Medical Research Council (mMRC) was used to assess the severity of dyspnea [4].

### 2.2 Operative Technique

**Figure 1:**
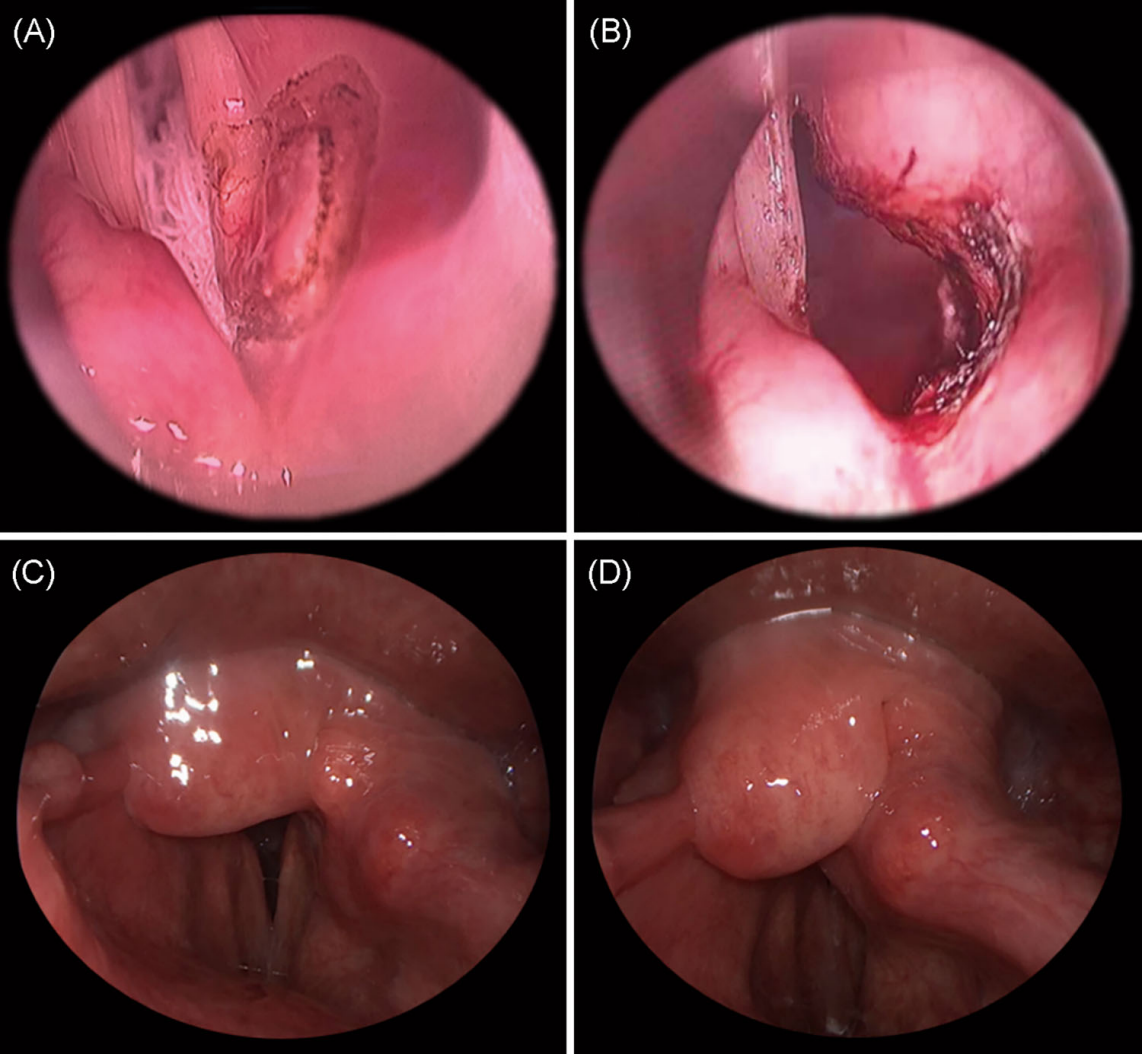
The intraoperative (A, B) and postoperative (C, D) laryngostroboscopy images of a 48‐year‐old male patient with bilateral vocal fold paralysis. (A, B) Intraoperative images captured during the right‐side modified arytenoidectomy. (C, D) Follow‐up laryngostroboscopy images 6 months post‐surgery. Panel C depicts the inspiratory phase, while panel D shows the phonatory phase.

All surgeries were performed by the same experienced surgeon using a CO_2_ laser under microscope‐guided suspension laryngoscopy. The procedure was performed on the immobile vocal fold, and if both sides were equally immobile, the right side was selected for convenience. The specific surgical steps are as follows: (1) The patient was positioned supine under general anesthesia. For non‐tracheostomy cases, a tracheal tube was inserted with a diameter of 6 mm for males and 5.5 mm for females, to a depth of 23–24 cm and the cuff was inflated with saline. (2) A laser laryngoscope was inserted via the oral cavity to expose the operative vocal fold and the arytenoid cartilage area, including the pyriform sinus. A wet gauze was inserted below the vocal fold to protect the trachea. The procedure was performed under a Zeiss OPMI Vario S88 microscope. Before the surgical procedure commenced, a laryngeal probe was used to define the surgical boundaries. The external boundary was the medial border of the thyroid cartilage; the anterior boundary was the vocal process of arytenoid cartilage; and the posterior boundary was the upper margin of the lamina of cricoid cartilage. (3) CO_2_ laser (AcuPulse Lumenis) was utilized with a power setting of 4.5–6 W, a spot size of 0.4 mm, a focused beam, and a continuous superpulse mode. The pulse duration was 0.4 s with a pulse interval of 0.8 s. During the procedure, the anesthesia was adjusted to 30% oxygen, with intermittent pure oxygen administration throughout the procedure. (4) The laser was used to incise the anterior margin of the vocal process and the attachment site of the vocal fold. The excision of the soft tissue obstructing the respiratory segment of the vocal folds, along with a partial resection of the arytenoid cartilage, was performed following the superior margin of the cricoid cartilage lamina. A probe was utilized during the procedure to assess the edge of the cricoid cartilage, ensuring the preservation of the airway profile while minimizing the risk of damage to the posterior mucosa of the posterior glottic commissure and the mucosa of the inner wall of the pyriform sinus (Figure 1A,B).

#### 2.2.1 Key points

**Figure 2:**
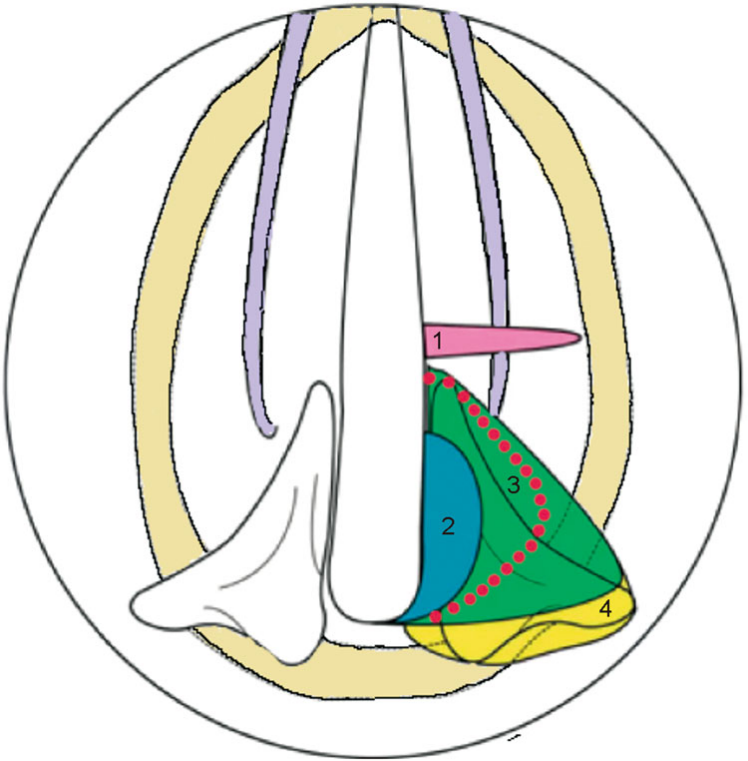
Top‐down view diagram of the laryngeal cartilage and schematic of the resection range of the arytenoidectomy. The red dashed line indicates the surgical resection area of the unilateral modified arytenoidectomy.

If both sides show similar immobility, the right side was selected due to the surgeon's right‐handed preference, which makes the procedure more convenient. Based on animal model results, Stages 2–3 were found to maintain glottic airflow while having minimal impact on voice quality (Figure 2). The “unilateral arytenoid contouring” technique was defined, focusing on the fovea triangularis cartilaginis arytaenoid. The medial and inferior surface of the arytenoid cartilage is polished as smoothly as possible, aligning with the medial edge of the cricoid cartilage, and transitioning gently toward the trachea, thereby expanding the airflow space beneath the arytenoid cartilage. This approach increases the space below the arytenoid cartilage for better airflow while preserving key structures such as the thyroarytenoid muscle and pyriform sinus mucosa. The apex of the arytenoid cartilage, the medial wall of the pyriform sinus, and the muscular process of the arytenoid cartilage are preserved to prevent the risk of the pyriform sinus mucosa collapsing and to avoid complications such as aspiration or choking during swallowing. Six hours postoperation, patients were instructed to begin oral hydration. If no swallowing difficulties, aspiration, or choking occur within 24 h, oral feeding is permitted. For non‐tracheostomized patients, postoperative monitoring in the ICU is conducted for 24 h. For those without contraindications to corticosteroid use, steroids are administered prophylactically to prevent laryngeal edema.

### 2.3 Statistical Analysis

Data analysis was performed using SPSS Statistics 27.0 and Wilcoxon rank‐sum test. Graphs were generated using GraphPad Prism 9.0.

## 3 Results

**Figure 3:**
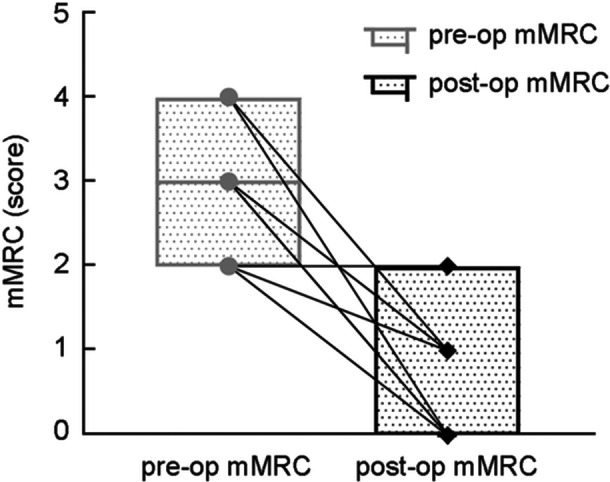
Preoperative and postoperative modified Medical Research Council (mMRC) scores, *n* = 19, *p* < 0.001.

**Figure 4:**
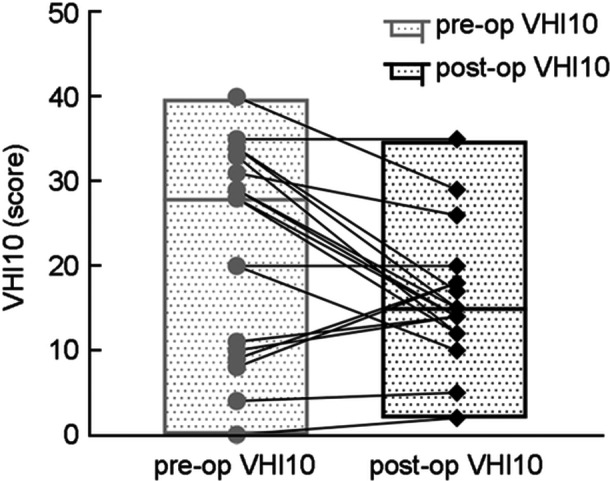
Preoperative and postoperative Voice Handicap Index‐10 (VHI‐10) scores, *n *= 19, *p* = 0.033.

Analysis of postoperative data at 6 months revealed improvements (Table 2 and Figures 3 and 4). Significant differences were observed in VHI‐10, mMRC, and Shimmer. Postoperative GRBAS scale data showed statistical differences in overall severity (G), roughness (R), and strain (S). Breathiness (B) and asthenia (A) did not show significant differences. All patients successfully removed mental tracheostomy tube postoperatively. Follow‐up for 6 months or longer revealed no recurrence of dyspnea. Postoperative evaluations showed no complaints of swallowing difficulties, aspiration, choking, or other clinical manifestations.

**Table 2:** Subjective and objective voice assessment (*n* = 19, mean ± SD).

Items	Preoperatively	Postoperatively	*p* value
Grade	2.56 ± 0.73	1.78 ± 0.44	0.008
Roughness	2.44 ± 0.73	1.67 ± 0.50	0.020
Breathiness	2.11 ± 1.05	1.67 ± 0.50	0.102
Asthenia	1.33 ± 0.87	1.00 ± 0.00	0.257
Strain	1.44 ± 0.88	0.56 ± 0.73	0.046
F0 (Hz)	175.54 ± 50.72	190.36 ± 39.28	0.196
MPT (s)	4.69 ± 4.30	5.98 ± 3.24	0.098
Jitter (%)	6.12 ± 6.14	2.39 ± 3.77	0.090
Shimmer (%)	22.27 ± 11.29	13.02 ± 6.71	0.048

## 4 Discussion

Management strategies for BVFP have included early‐stage reversible interventions such as tracheostomy, laterofixation, botulinum toxin injection, voice therapy, and definitive irreversible surgical treatments in later stages [5, 6, 7, 8]. Literature reports suggest that the duration of conservative treatment following the initial diagnosis of BVFP ranges from 6 months to 1 year [9, 10]. Approximately 36.2% of BVFP patients require emergency tracheostomy, with an average intubation duration of 6–9 months [11]. Thyroidectomy is the most common cause of BVFP in this study; 16 out of 19 patients had BVFP post‐thyroidectomy, consistent with previous findings.

Surgical interventions for BVFP encompass a range of techniques, primarily aimed at improving respiratory function while preserving voice quality. Since Jackson's introduction of the external laryngeal approach for posterior cordotomy in 1922, the treatment strategies for BVFP have significantly evolved [12]. In 1984, Ossoff pioneered the use of endoscopic laser arytenoidectomy [13], which subsequently led to the development of procedures such as posterior transverse cordotomy and partial or total arytenoidectomy [14]. Furthermore, techniques such as laryngeal pacing and reinnervation have been utilized to enhance both respiratory and phonatory functions [15]. Recent studies by Paniello et al. have identified the potential of autologous stem cell transplantation to the posterior cricoarytenoid muscle to augment abduction function may potentially provide a promising direction for future therapeutic interventions [16, 17].

Studies by Ahmed et al. have shown that there is no significant difference in vocal quality between coblation and diode laser treatments, while CO_2_ laser has a relatively minimal impact on vocal function [18, 19, 20]. In prior research involving over 140 patients who underwent transoral CO_2_ laser surgery, only 4 required tracheostomy, demonstrating the advantage of performing the procedure without the need for tracheostomy [21]. The majority of patients were able to avoid tracheostomy, thereby reducing postoperative discomfort and simplifying postoperative care [21]. In the cohort of patients enrolled in this study, two had already undergone tracheostomy upon admission, eight underwent prophylactic tracheostomy intraoperatively, and nine did not require tracheostomy. For those who did not undergo tracheostomy, close monitoring in the ICU was mandated postoperatively.

Four distinct ex vivo canine laryngeal models were constructed using traditional irreversible surgical techniques [1]. After comprehensive consideration of both ventilatory and voice functions, the UMA (depicted by the red line in Figure 2) was selected as the preferred approach, providing empirical evidence from animal models to inform clinical procedure selection [1]. In a retrospective clinical study by Bosley et al., the surgical approach depicted in the blue region of Figure 2 was found to preserve partial phonatory function; however, the postoperative relief of dyspnea was limited, with a success rate of only 62.5% [22]. Previous studies on glottic enlargement surgeries, including posterior cordotomy, and partial or total arytenoidectomy reported extubation rates ranging from 83% to 97% [23, 24]. In contrast, the extubation rate in this study reached 100%. Prior literature indicates that the average extubation time for such procedures is typically 2–3 months [25], whereas the modified surgical approach in this study resulted in an average extubation time of approximately 1 month, representing a notable reduction. Recent studies have explored arytenoidectomy combined with mucosal flap formation [25], but the UMA described herein offers a more streamlined approach, reducing operative time under general anesthesia.

The excision range of the UMA includes the vocal process and the contouring of the triangular fovea of the arytenoid cartilage, while preserving the vertical height. The UMA involves the excision of the connection between the thyroarytenoid muscle and the muscular process of the arytenoid cartilage. The majority of the thyroarytenoid muscle, which attaches to the lateral aspect of the arytenoid cartilage, is preserved, thereby maintaining the function responsible for controlling vocal fold tension. The procedure also includes ‘arytenoid contouring’ in the posterior one‐third of the vocal fold cartilage and its subglottic structures, thereby expanding the subglottic space and ensuring adequate airflow for respiratory support. The VHI‐10 scores showed a statistically significant reduction postoperatively, indicating a marked improvement in patients' self‐perceived vocal function. Furthermore, the subjective voice assessments conducted by otolaryngologists indicated improvements in overall severity, roughness, and strain. Trends indicated improvement in breathiness and asthenia, although these were not statistically significant. Objective acoustic analysis revealed a statistically significant reduction in Shimmer, with improvements in MPT and Jitter, suggesting that the vocal function was effectively preserved. The minimal impact on voice function can likely be attributed to the precise control of the surgical resection boundaries [26]. The modified surgical technique preserves the anterior two‐thirds of the vocal folds and maintains the integrity of the cricoarytenoid joint surface. Postoperatively, complete vocal fold closure during phonation is achieved, which likely accounts for the minimal impact on phonatory function. Postoperative follow‐up revealed a significant improvement in dyspnea, with all patients successfully extubated. Preoperative mMRC scores, which averaged 2.79, decreased to 0.58 postoperatively, indicating a substantial improvement in patients' quality of life.

Previous studies have identified several complications following CO₂ laser surgery, including granulation tissue formation at the incision site, scar contracture leading to glottic stenosis, difficulty with extubation, and postoperative aspiration or choking. One patient in this study developed granulation tissue postoperatively, which was managed with conservative pharmacological treatment for 100 days. Misiolek et al. found that the pathology of granulomas was consistent with inflammatory granulation tissue, where the healing and inflammation often overlap. Furthermore, they found that preoperative tracheostomy may disrupt the healing process of laryngeal inflammation, leading to an increased incidence of postoperative complications. In contrast, patients who did not undergo tracheostomy experienced a significantly lower rate of complications [12].

## 5 Conclusions

This study successfully translated the results of early animal experiments into a clinically applicable technique. The innovative UMA ensures adequate airway patency while also contributing to a measurable improvement in voice function. Thus, the modified procedure represents a safe, minimally invasive therapeutic approach that balances preserving voice quality and ensuring satisfactory airway clearance. Additionally, follow‐up evaluations in a select cohort of patients revealed a progressive enhancement in vocal function over time. However, the limited sample size remains a notable limitation of this study, which warrants further investigation in future research to substantiate these findings.

## Author Contributions

**Lu Xing:** conceptualization, investigation, data curation, writing – original draft, writing – review and editing. **Shu‐Yi Zeng:** writing – review and editing. **Pei‐Yun Zhuang:** conceptualization, supervision, project administration.

## Ethics Statement

All procedures performed in this study involving human participants were in accordance with the ethical standards of the institutional research editorial boards and with the 1964 Helsinki Declaration and its later amendments or comparable ethical standards. Ethics No: XMULAC20220265.

## Conflicts of Interest

The authors declare no conflicts of interest.

## Data Availability

The data that support the findings of this study are available on request from the corresponding author.
